# The Universality of Cooperative Fluctuations in Glass-Forming Supercooled Liquids

**DOI:** 10.1021/acs.jpclett.5c03185

**Published:** 2025-11-17

**Authors:** Jürgen E. K. Schawe, Kylian Hallavant, Antonella Esposito, Jörg F. Löffler, Allisson Saiter-Fourcin

**Affiliations:** † Laboratory of Metal Physics and Technology, Department of Materials, 27219 ETH Zurich, 8093 Zurich, Switzerland; ‡ 27040 Université de Rouen Normandie, INSA Rouen Normandie, CNRS, Groupe de Physique des Matériaux UMR 6634, 76000 Rouen, France

## Abstract

Amorphous glasses are formed by rapid cooling of the melt to avoid crystallization. The vitrification temperature increases with increasing the cooling rate. The viscosity of the supercooled melt and the vitrification are related to the atomic or molecular mobility, which in turn is linked to the dynamic heterogeneities and the size of cooperative rearrangement regions (CRR). The temperature dependence of the average correlation length of the CRR, *ξ*, for organic compounds, polymers, chalcogenide glass formers and metallic glass formers with a fragility index *m* ≥ 40 was investigated. The results show an almost material-independent variation of *ξ* as a function of the reduced reciprocal temperature *T*_g_/*T*, where *T*_g_ is the glass transition temperature. This suggests that *ξ*(*T*_g_/*T*) is a universal function for all the investigated glass formers. It was also found that *ξ* and the activation energy of the α-relaxation are proportional to each other. Furthermore, relations between the macroscopic parameters of the glass-forming kinetics were identified.

## Graphical abstract

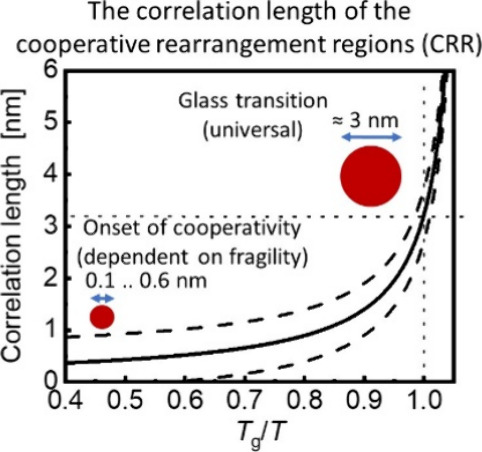

The dynamics in highly supercooled atomic or molecular structural glass-forming liquids are characterized by spatiotemporal heterogeneities that describe domains with differences in their atomic or molecular mobility.^1–5^ A classical thermodynamic theory using dynamic domains was developed by Adam and Gibbs,^6^ which describes the characteristic relaxation time, *τ*, in supercooled liquids as

1 $$\ln\tau \propto \frac{C}{TS_{c}}$$

where *S*_c_ is the configurational entropy, *T* is the macroscopic temperature, and *C* is a constant containing the free energy barrier of a rearrangement and the critical configuration entropy of the cooperative rearrangement regions (CRR). This thermodynamic approach is controversially discussed,^2,7–10^ but it was the first that allowed the description of both the local dynamic heterogeneities in supercooled liquids and the macroscopic kinetics of the glass transition. This theory thus laid the foundation for further investigations.

The CRR can be interpreted as dynamical spatiotemporal domains, and there are several models on the structure and composition of these domains.^11–13^ The complex spatial and dynamic structure of highly supercooled liquids is widely discussed because of its importance in understanding glass formation and related properties.^1,14–19^

The dynamic heterogeneities associated with the CRR have been measured by various techniques, such as the rotation of probe molecules measured by time-resolved optical spectroscopy^20^ or dielectric spectroscopy,^21^ multidimensional nuclear magnetic resonance,^22^ photon correlation and dielectric relaxation spectroscopy,^23,24^ light scattering,^25^ electron correlation microscopy,^26^ and quasi-elastic neutron scattering.^14^ A method for evaluating the relaxation function was described by Pieruccini and Alessandrini,^27^ and various techniques were proposed for determining the average characteristic value of the CRR. Takahara and others^10,28^ directly applied the Adam–Gibbs theory, where the critical configurational entropy was taken as the almost constant value of *S*_c_ at high temperatures in the melt, assuming that the cooperative domains contain one molecule. Another thermodynamic approach was developed by Donth^29,30^ based on the temperature fluctuations considered by the von Laue thermodynamics^31^ and linear response theory. In the classical Adam–Gibbs theory, the CRR are defined as the smallest spatial regions that can change their configuration independently of the neighboring subsystems. However, this definition does not reflect on the complex dynamics in the supercooled melt. We have therefore defined the CRR as the smallest representative thermodynamic subsystems that exhibit the same average temperature fluctuation as is characteristic for the α-relaxation of the macroscopic sample. In this approach, *ξ* is the average characteristic size of the subsystems representative of the α-relaxation, which are interpreted as the characteristic dynamic unit of the cooperative rearrangements. *ξ* can be estimated from the experimentally determined temperature fluctuation δ*T* caused by the α-relaxation, which represents the relaxation spectrum and thus not only the fast-dynamic clusters (as frequently discussed^11^), but also slowly moving regions. According to von Laue, the average temperature fluctuation of the smallest representative subsystem is

2 $$\delta T^{2}\approx k_{B}T^{2}\left(\frac{1}{C_{p,g}}-\frac{1}{C_{p,l}}\right)$$

where *k*_B_ is the Boltzmann constant, *T* is the mean temperature, and *C*_*p*,g_ and *C*_*p*,l_ are the heat capacities of the subsystem at temperatures below and above the α-relaxation transformation. The temperature fluctuation responsible for the α-relaxation is controlled by the change in temperature modulus *T*/*C*_*p*_ during the transformation. Since the heat capacity of the subsystem depends on its volume, this approach defines the volume of the CRR, which was recently verified experimentally via neutron scattering.^14^ In thermodynamic approaches, where the relationship between the relaxation spectrum and the temperature fluctuation is neglected, the correlation lengths obtained will be too large.^14,32^ The aforementioned approach has the advantage that it can be applied in a simple way to almost any glass former, which allows determining the temperature dependence of *ξ* over a wide temperature range. The limitation is that this technique can only report on the average magnitudes and average fluctuations of the relevant properties for subsystems involved in the α-relaxation and vitrification. We further assume that only dynamic heterogeneities contribute to the configurational or dynamic heat capacity.

In contrast to other definitions of the correlation length with respect to the dynamic structure of supercooled fluids, *ξ* here characterizes both the dimensions of the heterogeneities and the average dimension of the representative subsystems, which on average represents the entire spectrum of the α-relaxation, including the low-frequency side.

In computer simulations, string-like clusters are directly associated with the CRR,^8,33^ whereas compact structures are observed when the dynamic heterogeneities near the glass transition are experimentally visualized.^26^ This discrepancy may be explained by an averaging of the fast-moving string clusters in experiments. Another explanation could be that the fast-dynamic string clusters change into more compact structures as temperature decreases.^34^ The critical temperature to form such compact clusters of fast-moving particles is about 1.4 *T*_g_. Compact mobile clusters have also been proposed by molecular dynamics computer simulations using a different approach.^35^

All the above-mentioned different experimental techniques and evaluation approaches yield characteristic sizes of the dynamic heterogeneities in the supercooled liquid near the glass transition in the range of 1 to 5 nm. Recently, the temperature dependencies of the correlation length of two polymeric glass-formers and one atomic system with comparable macroscopic kinetics of glass formation were compared, and no influence of the molecular or atomic structure on *ξ* was found.^36^ The aim of this work is to compare the temperature dependencies of the characteristic length *ξ* of the dynamic heterogeneities for a wider range of different glass-formers, including low-molecular-weight organic compounds, polymers, chalcogenides, and metallic glass formers. In addition, the controversially discussed correlation between chemical structure, size of the dynamic heterogeneities, and macroscopic kinetics of the glass transition^25,30,33,36–40^ is investigated. The results reveal an almost universal behavior of *ξ*(*T*) for the investigated glass formers.

The glass transition has two main features. The first one is the dynamic atomic or molecular relaxation process in structurally equilibrated supercooled liquids. This is measured by spectroscopic techniques that determine the average relaxation time *τ* (or the relaxation frequency *ω*_r_ = *τ* ^–1^) and the related relaxation spectrum of the cooperative atomic or molecular motions.^37^ This process is usually referred to as α-relaxation or sometimes “dynamic glass transition”. The relaxation spectrum is often described by the Fourier-transform relaxation function, *ϕ*(*t*), containing a stretched exponential

3 $$\phi \left(t\right)=\exp\left(-{\left(\frac{t}{\tau_{k}}\right)}^{\beta_{K}}\right)$$

where *τ*_k_ is the relaxation time and *β*_K_ is the Kohlrausch exponent. The mean relaxation time *τ* ≈ 1/*ω*_max_ is determined from the frequency of the maximum of the dispersion peak. The difference between *τ* and *τ*_k_ is discussed in detail elsewhere.^41^ The mean relaxation time is representative of the mean mobility, which in turn is related to the spatially and temporally fluctuating association of particles, i.e. the dynamic domains or the CRR. The increase in relaxation time with decreasing temperature goes along with an increase in the size of the CRR.^6,42,43^

The temperature dependence of τ in the supercooled liquid below a cooperative onset temperature *T*_x_ in the melt^44–46^ follows the empirical Vogel–Fulcher–Tammann–Hesse (VFTH) equation^47–49^

4 $$\log\left(\frac{1}{\tau }\right)=A-\frac{B}{T-T_{v}}$$

where *A* and *B* are fitting parameters, and *T*_v_ is the VFTH temperature.

The second feature of the glass transition is vitrification (also known as the “thermal glass transition”), which occurs during cooling of the supercooled melt. This is a transformation of the structurally equilibrated supercooled melt into a structurally nonequilibrated glass. The cooperative atomic or molecular motions slow down during vitrification. This means that the cooperative rearrangements freeze, and consequently the configuration entropy becomes almost temperature-invariant during further cooling. The heat capacity decreases accordingly and the temperature dependence of the relaxation time turns from a VFTH behavior to an Arrhenius-like behavior.^50^ During this process, the supercooled liquid freezes into a glassy state. The relaxation time that characterizes the vitrification process depends on the cooling rate *β*_c_.^51,52^ Indeed, the liquid-to-glass transformation occurs at higher temperatures when higher cooling rates are used. The cooling-rate dependence of the vitrification temperature follows the VFTH equation as well

5 $$\log\;\left|\beta_{c}\right|=A_{c}-\frac{B}{T_{f}-T_{v}}$$

where *T*_f_ is the fictive temperature of vitrification at the cooling rate *β*_c_.

For thermo-rheologically simple materials (supercooled liquids with almost temperature-invariant shape of the α-relaxation spectrum), the reciprocal characteristic relaxation time at the temperature *T*_β_ is proportional to the cooling rate at which the material vitrifies at *T*_β_:

6 $$\left|\beta_{c}\right|\tau =C$$

where *C* is the proportionality constant.^52^ The consequence is that *B* and *T*_v_ in eqs 4 and 5 are identical, and *A*_c_ = *A* + log *C*. A more detailed discussion on the relation between vitrification and α-relaxation is given in ref 52.

A normalized property widely used to characterize the kinetics of the glass transition is based on the fragility concept introduced by Angell.^53^ The fragility index *m* is defined as the normalized slope of the curve log­(1/*τ*) or log­(|*β*_c_|) versus the reciprocal temperature 1/*T* at the glass transition temperature *T*_g_. Assuming the validity of the VFTH equation, the fragility index can be expressed as

7 $$m=-{\frac{d\left(\log\left|\beta_{c}\right|\right)}{d\left(\frac{T_{g}}{T}\right)}|}_{T=T_{g}}=\frac{BT_{g}}{{\left(T_{g}-T_{v}\right)}^{2}}$$

where minor differences of the fragility index between relaxation and vitrification measurements are due to differences in the definition of the glass transition temperature. The fragility index starts at a value of 16 and can reach values above 200.^54^ The lowest value corresponds to an Arrhenius behavior. Supercooled liquids with a low fragility index are classified as strong glass-formers, whereas high values of the fragility index characterize fragile glass-formers.

The glass transition temperature, *T*_g_, is often defined as the temperature at which the relaxation time in the supercooled liquid is between 100 and 1000 s,^55^ or as the temperature at which the sample vitrifies during cooling at 10 K/min. This corresponds to 1.2 ≤ log *C* ≤ 2.2.

During vitrification, the atomic or molecular motions slow down and the relaxation time increases as temperature decreases. This leads to an increase in the width of the transition region during cooling, Δ*T*_β_, compared to the transition width, Δ*T*_ω_, measured in the structurally equilibrated supercooled liquid.

The glass transition temperatures of the materials from Table 1 with known temperature dependencies of the average CRR correlation length have a fragility index of 40 < *m* < 220. Orientational, spin and colloidal glass-formers are not considered. The measured temperature range is between 160 and 580 K. Most of the data presented in this work are obtained by heat capacity spectroscopy or are based on the cooling-rate dependence of the vitrification process. These techniques provide comparable information on the dynamics in supercooled glass-forming liquids.^52^ To identify correlations for these results over such a wide range, *ξ* is plotted versus the reduced reciprocal temperature *T*_g_/*T* (Figure 1). First of all, it can be seen that the correlation length increases with the reduced reciprocal temperature for all the considered materials. This observation is in agreement with previous reports.^24,36^ No evidence of a constant or decreasing value of *ξ* with reduced reciprocal temperature, as occasionally reported,^25^ was found for these data. This may result from the fact that stronger glass-formers (*m* < 40) show less pronounced changes of *ξ* with temperature.

**Table 1:** Literature Data of Glass-Forming Liquids for Which the Temperature Dependence of the Correlation Length Was Reported^a^

Number	Material	*T*_g_ [K]	*m*	*B* [K]	*T*_v_ [K]	*ξ*(*T*_g_) [nm]	Reference
1	PPglycol	167	48	881	112.5	3.857	^82,83^
2	PIB	209	43	1154	131	2.92	^82,84^
3	Glycerol	193	49	1085	128	3.53	^82,84^
4	SBR	215	97		184	2.7	^82,85^
5	BIBE	220	100	724	180	4.42	^86^
6	EVA_60	248	89	804.5	190.6	2.4	^87^
7	EVA_80	276	92	915	209.9	2.6	^87,88^
8	PnBMA2%	303	70	318	266		^82,89^
9	PnBMA19%	306	72	323	269		^82,89^
10	Se	310	81	652	260	3.3	^36^
11	PVAc	312	94	608	267	3.046	^36^
12	PBF	311	111	548	271	3.42	^90^
13	PEMA	338	87	1620	261.7		^14,91^
14	PPT	320	150	409.2	283.9	3.132	^90^
15	PPF	330	111	584.7	289.4	3.38	^90^
16	PLA	327	139	580	290	3.59	^36^
17	2,4-PEF	343	125	534	300	3.6	^92^
18	PE-2,4–2,5F	349	118	581	304	3.3	^92^
19	2,5-PEF	356	116	584.9	304.4	3.15	^90^
20	PETg	351	151	427	317	3.147	^32^
21	PET	353	160	421.6	314	4.31	^90^
22	PMMA	377	136	185	354.3	1.53	^82,93^
23	PVC	357	191	357	329	2.8	^32^
24	PS	373	117	475.3	334.4	3.45	^82,93^
25	PC	417	151	891	367	2.98	^32^
26	PEI	489	162	599	442	3.4	^32^
27	Pt-based glass	498	80	2310	408	2.80	^60^
28	Pd-based glass	576	69	1534	463	2.65	^60^

a The full names of the materials are listed in Table S1 of the Supporting Information (SI).

**Figure 1:**
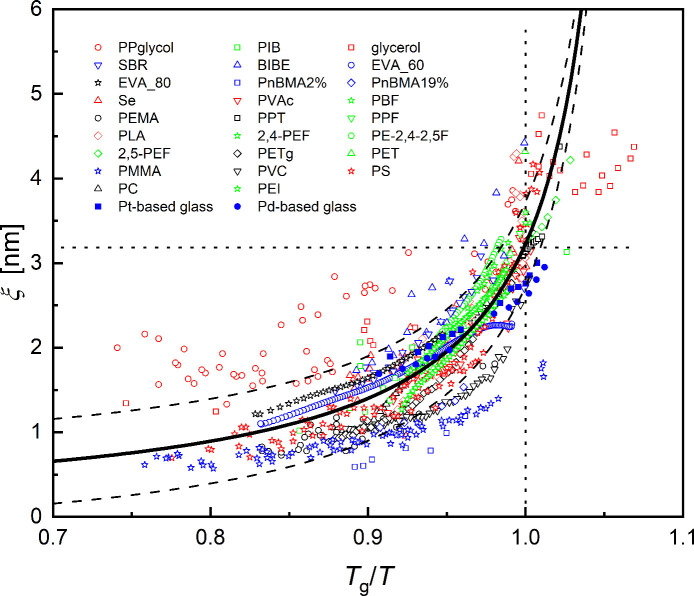
Average size of the CRR as a function of the reduced reciprocal temperature *T*_g_/*T*. The values were obtained with the support of the literature. The references and list of materials are presented in Table 1 and Table S1 of the SI.

The majority of the data fluctuates at scales of about ± 0.5 nm around the average black solid curve in Figure 1. This fluctuation is represented by the dashed curves in Figure 1, which illustrate the approximate experimental uncertainty of the collected data. The literature data on propylene glycol show the largest fluctuation. The upper outlier with the largest values of *ξ*(*T*) results from the photoinitiator benzoin isobutylether (BIBE). The reason for this is unknown. The lower outlier results from poly (methyl methacrylate) (PMMA). According to eq 17 in Methods, this polymer has a relatively low CRR correlation length. A reason for this could be the impact of a second relaxation process (influenced or caused by the side chains) on the glass transition process, which seems to be typical of polyacrylates.^56–58^ This may influence the shape of the glass transition and therefore lead to lower values of *ξ*(*T*_g_). An evaluation based on the boson peak spectrum led to *ξ*(*T*_g_) = 2.54 nm,^59^ which agrees well with the correlation length of the other polymers.

The average of all data in Figure 1 is marked by the black solid curve. This temperature dependence of *ξ* is described by

8 $$\xi {\left(T\right)}^{-1}=\xi_{0}^{-1}-\left(\xi_{0}^{-1}-\xi {\left(T_{g}\right)}^{-1}\right)\frac{T_{g}}{T}$$

where *ξ*_0_ is the correlation length extrapolated to high temperatures. This property and *ξ*(*T*_g_) are the only fitting parameters. *ξ*_0_ can be interpreted as the onset correlation length for cooperative rearrangements at the cooperative onset temperature *T*_x_, whereas *ξ*(*T*_g_) is the average CRR correlation length at *T*_g_. There is some evidence that the shape of the CRR near *T*_x_ is not spherical,^34^ thus this must be considered as a value where the volume of the CRR is normalized to a spherical dimension. By considering all the data plotted in Figure 1, the black solid curve corresponds to *ξ*_0_ = 0.2 nm and *ξ*(*T*_g_) = 3.2 nm.

While the relaxation time between the onset of α-relaxation and the glass transition changes by several orders of magnitude (typically 8 decades), the CRR correlation length does not change by much more than 1 order of magnitude.

The relatively small material-dependent variations in the measured data of *ξ* indicate a universality of the temperature dependence of *ξ* with respect to the reduced reciprocal temperature *T*_g_/*T* and the CRR size at *T*_g_. For the Pt- and Pd-based metallic glass-formers in Table 1, it has been shown that the CRR correlation length depends linearly on the apparent activation energy of the vitrification process.^60^ This indicates that the non-Arrhenian behavior of the glass transition is caused by an increase in CRR size with decreasing temperature. The universality of the correlation length behavior agrees well with the results of numerical simulations by Wang et al.,^33^ who also compared metallic and polymeric glass-formers. The universality of the temperature dependence of ξ also seems to be the reason for a macroscopic universality of the glass transition kinetics, as discussed by Rössler et al.^61^

The individual curves of *ξ*(*T*) were fitted with eq 8, and the resulting values of *ξ*_0_ and *ξ*(*T*_g_) are listed in Table S1 in the Supporting Information (SI). A first question may be raised regarding the dependence of the correlation length at *T*_g_ on the kinetics of the glass transition. Neglecting the data on PMMA, we can approximate *ξ*(*T*_g_) to be around 3.2 nm for all the investigated glass-formers. In this discussion, only atomic or molecular structural glass-formers with a fragility index above 40 were considered, which means that very strong glass-forming materials are not incorporated due to a lack of data.

A comparison of the expected temperature dependence of the CRR correlation length *ξ* with measurements of the correlation length of dynamic heterogeneities with comparable mobility, *ξ*_dh_,^26^ shows that *ξ* ≈ 2*ξ*_dh_. This discrepancy between correlation lengths is consistent with the assumption that a CRR contains domains with high and low mobility,^13^ and that the interaction of both components has a significant contribution to the α-relaxation spectrum.

The kinetic properties *m* and *T*_g_ depend on the atomic or molecular structure and on the chemical composition of the glass-forming liquid.^62–64^ In the case of a correlation between *m* and/or *T*_g_ and *ξ*(*T*_g_), one may conclude that the correlation length at *T*_g_ also depends on the atomic or molecular structure and chemical composition. However, the data of *ξ*(*T*_g_) in Figure 2a show no correlation with the fragility index *m*, which is in agreement with the literature.^25,30,36,59,65^ Polyacrylates stand out with their peculiar behavior, as discussed previously (see also ref.^30^). The values of the correlation length also show no clear dependence on the glass transition temperature, with the exception of the polyacrylates (Figure 2b). This indicates that the molecular or atomic structure has no significant (or just a minor) influence on the CRR size at *T*_g_. The specific size of the individual CRR at *T*_g_ is therefore submolecular (or on the atomic scale) in fragile glass-formers. In this case, the elementary unit may be related to the “beats” used to calculate thermodynamic properties.^66–68^ Furthermore, Figure 2 shows that the interaction between the α-relaxation and secondary relaxations (as in the example of polyacrylates) reduces the CRR size or affects the measurement of temperature fluctuations and therefore the measurement results.

**Figure 2:**
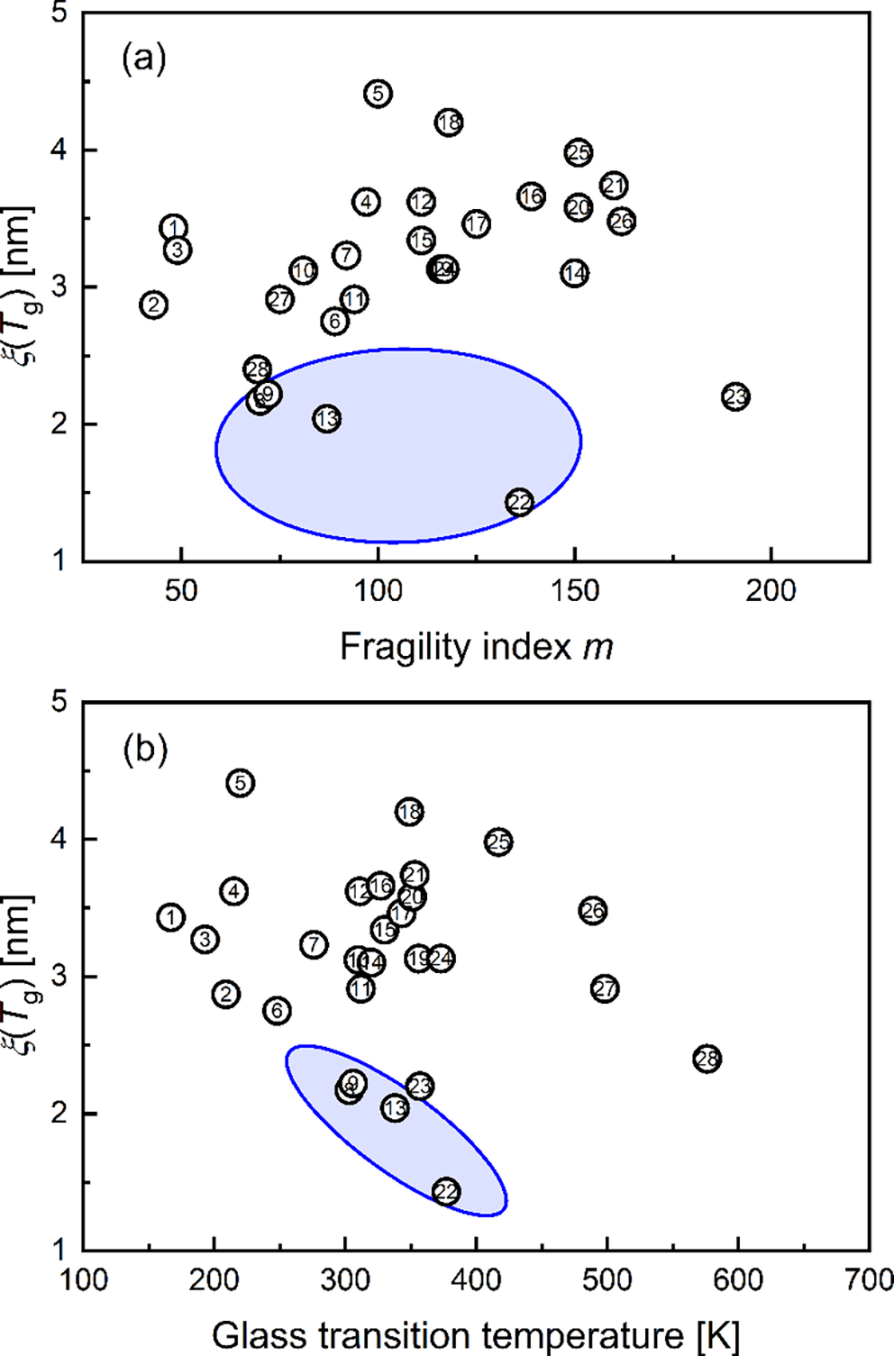
Correlation length of the CRR at the glass transition. *ξ*(*T*_g_) as a function of (a) the fragility index *m* and (b) the glass transition temperature *T*_g_. The regions of the polyacrylates (samples 8,9,13 and 22) are highlighted. The numbers in the figures correspond to the materials listed in Table 1 and Table S1 of the SI; the values of *ξ*(*T*_g_) are taken from Table S1 of the SI.

The function *ξ*(*T*) (eq 8) provides the CRR correlation length at the onset of cooperativity, *ξ*_0_, at *T*_x_ ≫ *T*_g_,^45,46^ which is expected to depend on the chemical composition and the molecular or atomic interactions. Usually *T*_x_ is reported to be between near the melting temperature and up to 2 *T*_g_.^69–72^ Significantly lower values are only found for a few organic compounds^73^ and copolymers.^46^

To investigate the relation between ξ_0_ and the kinetics of the glass transition, ξ_0_ was plotted versus the fragility index *m*. Figure 3 indicates a correlation according to which ξ_0_ increases with decreasing *m*. In particular, Figure 3 shows that ξ_0_ varies between 0.1 and 0.6 nm for the investigated range of *m*. For organic compounds and polymers, this value is approximately in the range between the next-neighbor atomic distance^74^ and the benzene–benzene center-of-mass distance of 0.5 nm.^75^ For the glass-forming metal melts, *ξ*_0_ is about 0.35 nm. This value corresponds to the size of the structural unit in a metal melt^76^ and is consistent with the characteristic length of medium-range order (MRO) in metallic glasses.^77^ We therefore hypothesize that the elementary units for cooperativity, which compose the CRR, coincide with the MRO in metallic glass. The correlation between the size of MRO and the fragility index discussed in ref 77 is essentially in agreement with the present findings on the correlation between *ξ*_0_ and *m*.

**Figure 3:**
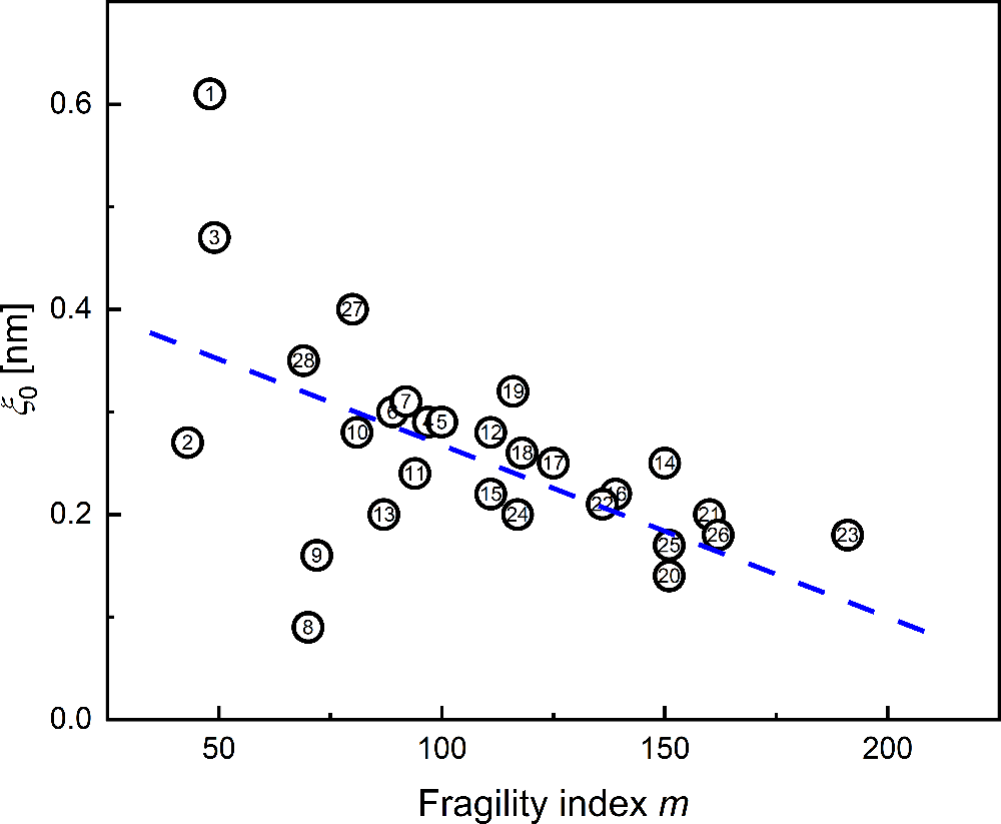
Size of the CRR at the onset of cooperativity. *ξ*_0_ as a function of fragility index *m*. The dashed line is a guide for the eyes. The numbers in the figures correspond to the materials listed in Table 1 and Table S1 of the SI; the values of *ξ*_0_ are taken from Table S1 of the SI.

The presented temperature dependence shows that the CRR correlation length changes by a factor of about 10 to 20 in the temperature range between *T*_x_ and *T*_g,_, and the length change *ξ*(*T*_g_) – *ξ*_0_ decreases with decreasing fragility index. In contrast, the viscosity, diffusion coefficient, and relaxation time change by about 10 orders of magnitude in this temperature range. Therefore, it is unlikely that *ξ* directly determines the relaxation time. Rather, a direct relationship is expected between the correlation length and the apparent activation energy required for CRR formation.

The apparent activation energy *E*_a_ can be directly determined from the VFTH equation

9 $$E_{a}=R\frac{d\left(\ln\tau \right)}{d\left(\frac{1}{T}\right)}=\ln10\;R\frac{BT^{2}}{{\left(T-T_{v}\right)}^{2}}$$

where *R* is the gas constant.

It is evident that *E*_a_ increases as the limiting temperature *T*_v_ is approached. For the materials analyzed in Figure 1, *E*_a_ is plotted as a function of *ξ* in Figure 4a. Within the order of experimental uncertainty, this relationship is linear through the origin:

10 $$E_{a}=\sigma \;\xi$$

where *σ* is the constant slope that describes the increase in activation energy with increasing CRR correlation length. This suggests that the average size of the CRR essentially controls the slope of the VFTH behavior, rather than the relaxation time.

**Figure 4:**
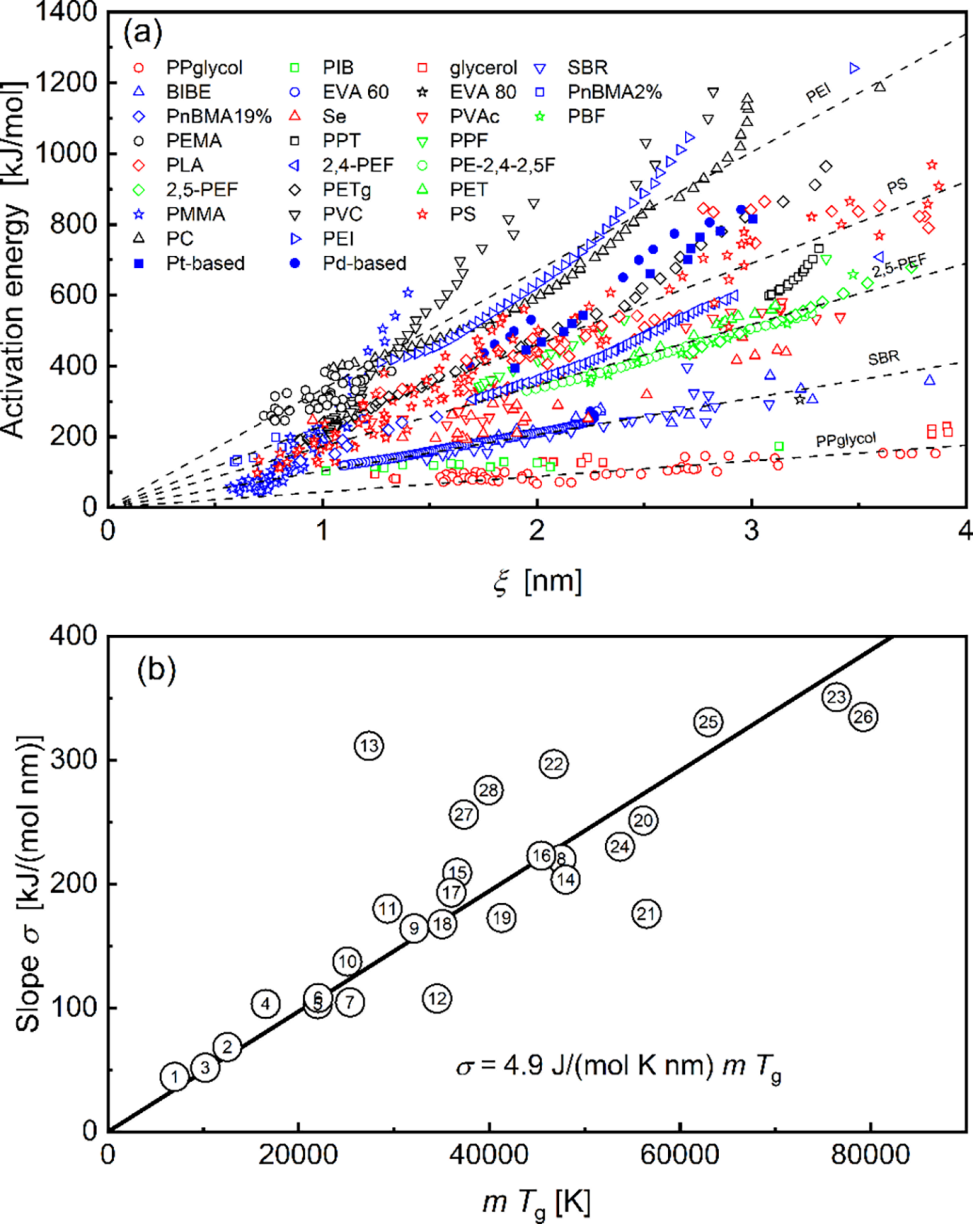
Relation between activation energy and CRR correlation length. (a) *E*_a_ as a function of *ξ*. The data points were fitted using eq 10. For clarity, the fitting results for only five materials are shown. (b) Slope of the linear fitting in Figure 4a as a function of the product of fragility index and glass transition temperature.

The comparison of eqs 7 and 9 leads to

11 $$m=\frac{1}{\ln10}\frac{E_{a}\left(T_{g}\right)}{RT_{g}}$$

The fragility index is thus proportional to the ratio of activation energy and thermal energy at the glass transition. By inserting eq 11 into eq 10 and rearranging, the slope *σ* is obtained:

12 $$\sigma =\frac{dE_{a}}{d\xi }\approx \frac{E_{a}}{\xi }=\frac{\ln10\;R}{\xi \left(T_{g}\right)}mT_{g}$$

If *ξ*(*T*_g_) is nearly constant, the slope σ should be proportional to *mT*_g_. The corresponding plot is shown in Figure 4b. The data are in good agreement with a linear behavior. The fit yields a proportionality factor of 4.9 J/(mol K nm), which leads to *ξ*(*T*_g_) = 3.9 nm and is in good agreement with the data presented in Figure 2. We interpret this result as an independent argument for the universality of *ξ*(*T*_g_) in the investigated range of fragility indices.

Considering the relation between δ*T* and *E*_a_ derived from the VFTH equation, it is shown in ref 60 and its related Supporting Information that a relation between *ξ* and *E*_a_ of the following form can be obtained from eq 17 displayed in Methods:

13 $$\xi \propto {\left(\frac{E_{a}}{T}\right)}^{2/3}$$

Within the order of experimental uncertainty, this equation does not differ significantly from the linear fits shown in Figure 4a. We thus understand the results in Figure 4b as an argument in favor of the linear approach discussed above. Additional theoretical arguments or additional experimental data are required to decide between the two approaches (eqs 10 and 13).

The relations between the empirical macroscopic kinetic parameters of the glass transition have been widely discussed.^54^ This work considers the relationship between the glass transition temperature obtained from the VFTH parameters, *T*_v_ and *B*, and the fragility index *m*, which are re-examined using the data in Tables 1 and 2. It turns out that the relationship is well described by

14 $$\frac{T_{v}}{T_{g}}=1-\frac{m_{0}}{m}$$

where *m*_0_ ≈ 16 is the minimum fragility index for the strongest glass formers.^78^ This equation agrees with the known boundary conditions that *T*_v_ = 0 K leads to *m* = *m*_0_ and that *m* increases strongly when *T*_v_ */T*_g_ tends to 1. Moreover, this parameter-free equation shows an excellent agreement with the experimental data (Figure 5a), especially considering the uncertainties expected from Figure S1 in the SI.

**Table 2:** Literature Data of Glass-Forming Liquids for Which the Temperature Dependence of the Correlation Length Was Not Reported

Number	Material	*T*_g_ [K]	*m*	*B* [K]	*T*_v_ [K]	Reference
29	DGG1^a^	809	42	4331.6	520.8	^94^
30	Fe_6_S_7_ (spin glass)^b^	7.1	17	83	1.2	^81^
31	Haplo-rhyolite	1108	23	15950	232.2	^79^
32	Phonolite	884	24	10261	263.8	^79^
33	Trachyte	935	25	10449	303.7	^79^
34	SiO_2_	1457	25	15336	508.5	^79^
35	Basalt	936	42	6101	567.0	^79^
36	55K_2_CO_3_-45MgCO_**3**_	476	64	1914	356.0	^79^
37	Peridotite	989	71	3703	761.7	^79^
38	Na_2_CO_3_	383	110	832	329.0	^79^
39	40CaNO_3_-60K_2_NO_3_	376	115	798	324.4	^79^
40	Mg_65_Cu_25_Y_10_	402	50	2495	260	^80^
41	Pd_40_Ni_40_P_20_	578	50	3066	390	^80^
42	Pd_48_Ni_32_P_20_	590	43	2826	392	^80^
43	Pd_77.5_Cu_6_Si_16.5_	637	73	23767	493	^80^
44	Cu_47_Ti_34_Zr_11_Ni_8_	673	59	26056	500	^80^
45	Zr_41.2_Ti_13.8_Cu_12.5_Ni_10_Be_22.5_	625	46	3314	412	^80^
46	Zr_46.75_Ti_8.25_Cu_7.5_Ni_10_Be_27.5_	590	46	3667	372	^80^
47	GeO_2_	816	21	9731	199	^80^

a The composition of DGG1 in weight % is SiO_2_ 71.72, Na_2_O 14.95, CaO 6.73, MgO 4.28, Al_2_O_3_ 1.23, SO_3_ 0.436, K_2_O 0.38, Fe_2_O_3_ 0.191, and TiO_2_ 0.137.

b The values were calculated from the data given in ref 81.

**Figure 5:**
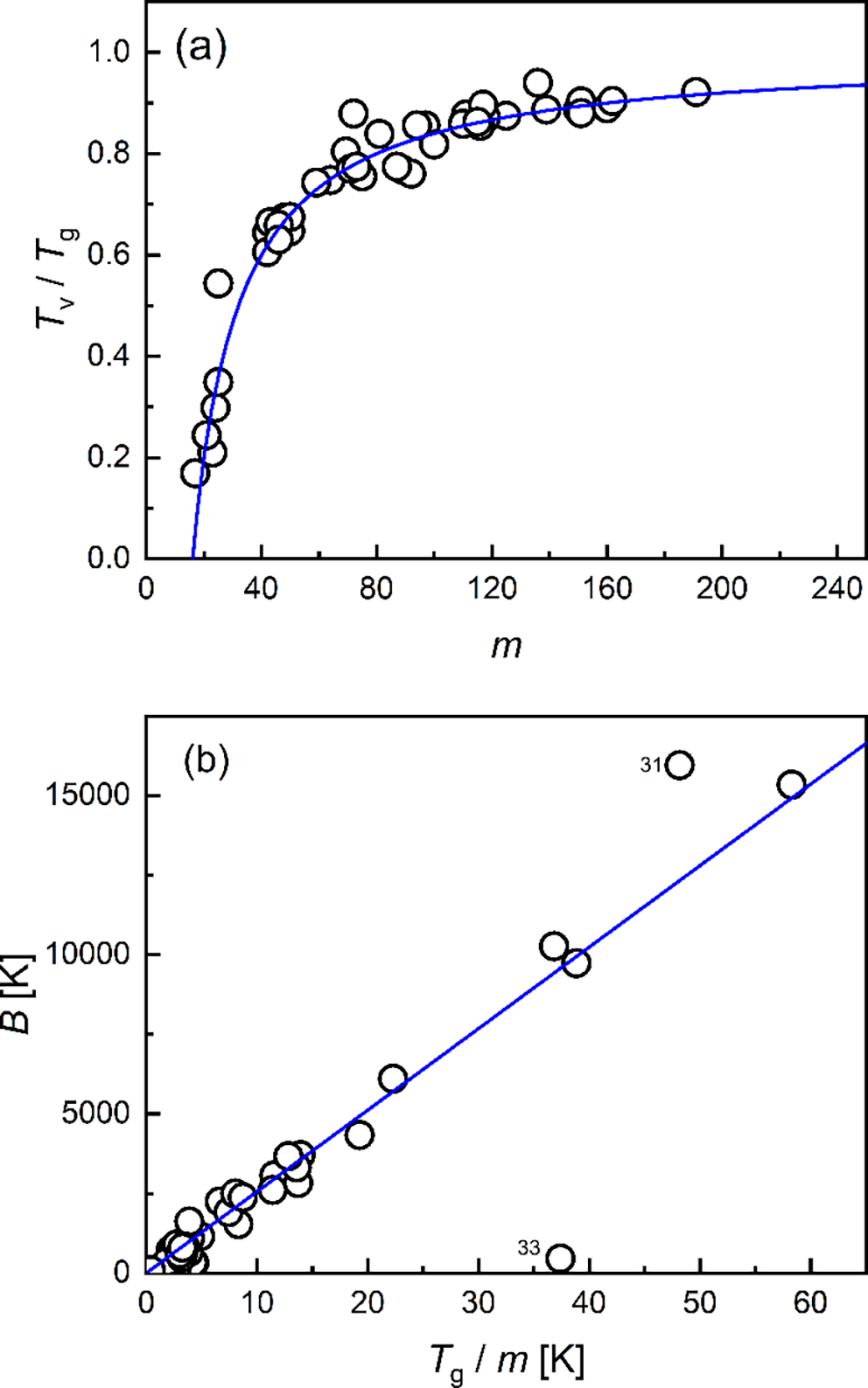
Relation between the VFTH parameters and the fragility index. Comparison of the data from Tables 1 and 2 with plots of (a) eq 14 and (b) eq 15. The two parameter-free equations are illustrated by blue solid curves.

Considering the relationship between *m* and the VFTH parameters (eq 7), another parameter-free relationship results from eq 14:

15 $$\frac{B}{T_{g}}=\frac{{m_{0}}^{2}}{m}$$

The comparison of this equation with the data from Tables 1 and 2 is shown in Figure 5b. Only the data for materials 31 (haplo-rhyolite) and 33 (trachyte) deviate from the expected behavior. This may be interpreted by a lack of consistency in the reported data.

Equations 14 and 15 show the relation between *T*_g_, *T*_v_, *B* and *m*. Only two of these parameters are needed to describe the kinetics in a supercooled liquid approaching the glass transition. In addition, these equations reveal the complementarity of other parameters used to describe fragility. For instance, considering the kinetic fragility parameter *K* = *T*_v_/*B*, the proportionality between *K* and *m* – *m*_0_ follows from eqs 14 and 15:

16 $$K=\frac{m-m_{0}}{{m_{0}}^{2}}$$

The reciprocal is the frequently used dynamic fragility parameter D*.

In conclusion, a universal behavior of the temperature dependence of the CRR correlation length *ξ* is proven for a wide set of different structural glass-forming liquids with a fragility index *m* > 40. Based on this experimental data set, a phenomenological relation with two new parameters having direct physical meaning was introduced, namely the correlation length at the glass transition temperature, *ξ*(*T*_g_), and the correlation length at the onset of cooperativity, *ξ*_0_.

Within the order of magnitude of experimental variations, the correlation length at *T*_g_ is about 3.2 nm and decreases with increasing temperature. A significantly lower value is measured for PMMA, likely because additional motions of side groups in polyacrylates increase the overall mobility in the CRR or the measured temperature fluctuation. In general, no difference was observed between metals, low-molecular-weight glass formers, and polymers, contrary to the statements of ref 38. Consequently, *ξ*(*T*_g_) is an almost universal property that does not significantly depend either on short-distance interactions or the size of the elementary relaxing units. A general difference between molecular systems, polymers, chalcogenide glass formers and glass-forming metal alloys was not found. The individual atomic or molecular sizes and the specific atomic/molecular interactions have thus no significant influence on the CRR size.

The minimum correlation length at high temperature, *ξ*_0_, decreases with increasing fragility index. This indicates the influence of chemical structure on the elementary unit of the cooperative movement. However, for molecular systems, the elementary unit is smaller than the molecule or monomer unit. This influences the determination of the number of particles in the CRR.

Furthermore, it is shown that ξ is in good approximation proportional to the apparent activation energy of the α-relaxation. This indicates that the temperature dependence of *ξ* controls the non-Arrhenian behavior.

In addition, the relations between the kinetic parameters *T*_g_, *T*_v_, *B* and *m* were analyzed for structural glasses in a fragility range between 21 and 190 and for a spin glass with *m* = 17. It is shown that these parameters are not independent from each other, and that two of them are sufficient to describe the macroscopic kinetics near *T*_g_.

## Methods

To study the relationships between the characteristic size of the CRR, the temperature of the supercooled liquid, and the macroscopic kinetics at the glass transition, the literature was searched for measurements of the temperature dependence of *ξ*, the VFTH parameters, and the fragility index *m*. Comparison of these data identifies some minor discrepancies that could lead to increased errors in Figures 2a, 3, and 4 for these materials (see SI). The materials include low-molecular-weight organic compounds, polymers, the simplest chalcogenide glass-former selenium, and two bulk glass-forming metal alloys (Table 1). The full names of the considered materials are listed in Table S1 of the SI. Other materials were also added to further complete the list, such as a silicon lime glass-former (DGG1), Si and Ge oxides, carbonate melts,^79^ further glass-forming metal alloys,^80^ and the pyrrhotite Fe_6_S_7_ as a spin-glass former with a very low glass transition temperature and very broad relaxation spectrum^81^ (Table 2).

For the materials in Table 1, the average cooperative length was determined according to the procedure introduced by Donth^29^ under the assumption that the CRR have a spherical shape, which can be assumed at least for a temperature range of *T*_g_/*T* > 0.7 ^34^

17 $$\xi ={\left(\frac{6}{\pi }\frac{k_{B}}{\rho }{\left(\frac{T_{f}}{\delta T}\right)}^{2}\left(\frac{1}{c_{p,g}}-\frac{1}{c_{p,l}}\right)\right)}^{1/3}$$

where δ*T* is the temperature fluctuation determined from the width of the glass transition, *c*_*p*,g_ and *c*_*p*,l_ are the specific heat capacities of the glassy and liquid states, *ρ* is the density, and *k*_B_ is the Boltzmann constant. For the determination of *ξ*, it is assumed that the supercooled liquid is isotropic. In this case, the average correlation length is independent of the direction and actual shape of the individual subsystems, and the model assumption of a sphere is the simplest solution. The validity of this approach was recently confirmed by quasi-elastic neutron scattering.^14^ Some data of *ξ*(*T*_g_) in Table 1 differ slightly from the values reported by Hong et al.,^59^ obtained from boson peak spectra.

## Supplementary Material



## Data Availability

The data sets used in the current study are available from the corresponding author on reasonable request.

## Notes

Consistency test of the used data, sample names and fitting results (PDF)

## References

[1] Tahaei A., Biroli G., Ozawa M., Popović M., Wyart M. (2023). Scaling description of dynamical deterogeneity and avalanches of
relaxation in glass-forming liquids. Phys. Rev.
X.

[2] Berthier L. (2021). Self-Induced
Heterogeneity in Deeply Supercooled Liquids. Phys. Rev. Lett..

[3] Berthier L., Reichman D. R. (2023). Modern computational studies of the glass transition. Nat. Rev. Phys..

[4] Adhikari A. N., Capurso N. A., Bingemann D. (2007). Heterogeneous
dynamics and dynamic
heterogeneities at the glass transition probed with single molecule
spectroscopy. J. Chem. Phys..

[5] Kirchner K. A. (2023). Beyond the Average: Spatial and Temporal Fluctuations in Oxide Glass-Forming
Systems. Chem. Rev..

[6] Adam G., Gibbs J. H. (1965). On the temperature
dependence of cooperative relaxation
properties in glass forming liquids. J. Chem.
Phys..

[7] Dyre J. C., Hechsher T., Niss K. (2009). A brief critique
of the Adam-Gibbs
entropy model. J. Non-Cryst. Solids.

[8] Starr F. W., Douglas J. F., Sastry S. (2013). The relationship
of dynamical heterogeneity
to the Adam-Gibbs and random first-order theories of glass formation. J. Chem. Phys..

[9] Ozawa M., Scalliet C., Ninarello A., Berthier L. (2019). Does the Adam-Gibbs
relation holds in simulated supercooled liquids?. J. Chem. Phys..

[10] Tatsumi S., Aso S., Yamamuro O. (2012). Thermodynamic
study of simple molecular glasses: Universal
features in their heat capacity and the size of the cooperatively
rearranging regions. Phys. Rev. Lett..

[11] Ediger M. D. (2000). Spatially
heterogeneous dynamics in supercooled liquids. Annu. Rev. Phys. Chem..

[12] Sillescu H. (1999). Heterogeneity
at the glass transition: a review. J. Non-Cryst.
Solids.

[13] Donth E. (1999). Phenomenological
treatment of dynamic glass transition heterogeneity. Acta Polym..

[14] Chua Y. Z. (2023). Determination of Cooperativity
Length in a Glass-Forming Polymer. ACS Phys.
Chem. Au.

[15] Paul K., Mutneja A., Nandi S. K., Karmakar S. (2023). Dynamical heterogeneity
in active glasses is inherently different from its equilibrium behavior. Proc. Natl. Acad. Sci. U.S.A..

[16] Royall C. P., Turci F., Speck T. (2020). Dynamical
phase transitions and their
relation to structural and thermodynamic aspects of glass physics. J. Chem. Phys..

[17] Jug G., Loidl A., Tanaka H. (2021). On the structural
heterogeneity of
supercooled liquids and glasses. EPL.

[18] Vila-Costa A. (2023). Emergence of equilibrated
liquid regions within the glass. Nat. Phys..

[19] Ruiz-Ruiz M. (2023). Real-time microscopy
of the relaxation of a glass. Nat. Phys..

[20] Cicerone M. T., Blackburn F. R., Ediger M. D. (1995). How do molecules move near Tg? Molecular
rotation of six probes in o-terphenyl across 14 decades in time. J. Chem. Phys..

[21] van
den Berg O., Wübbenhorst M., Picken S. J., Jager W. F. (2005). Characteristic
size of molecular dynamics in polymers probed by dielectric probes
of variable length. J. Non-Cryst. Solids.

[22] Reinsberg S. A. (2001). Length scale of dynamic
heterogeneity in supercooled glycerol near *T*
_g_. J. Chem. Phys..

[23] Rizos A. K., Ngai K. L. (1999). Experimental determination
of the cooperative length
scale of a glass-forming liquid near the glass transition temperature. Phys. Rev. E.

[24] Cangialosi D., Alegría A., Colmenero J. (2008). Dielectric relaxation of polychlorinated
biphenyl/toluenemixtures: Component dynamics. J. Chem. Phys..

[25] Hong L., Novikov V. N., Sokolov A. P. (2011). Dynamic
heterogeneities, boson peak,
and activation volume in glass-forming liquids. Phys. Rev. E.

[26] Zhang P. (2018). Spatially heterogeneous
dynamics in a metallic glass forming liquid
imaged by electron correlation microscopy. Nat.
Commun..

[27] Pieruccini M., Alessandrini A. (2015). Method for
estimating the cooperativity length in polymers. Phys. Rev. E.

[28] Takahara S., Yamamuro O., Matsuo T. (1995). Calorimetric
study of 3-bromopentane:
Correlation between structural relaxation time and configurational
entropy. J. Phys. Chem..

[29] Donth E. (1982). The size of
cooperatively rearranging regions at the glass transition. J. Non-Cryst. Solids.

[30] Hempel E. (2000). Characteristic length of dynamic glass transition near
Tg for a wide
assortment of glass-forming substances. J. Phys.
Chem. B.

[31] von
Laue M. (1917). Temperatur- und Dichteschwankungen. Phys. Zeitschr..

[32] Rijal B., Delbreilh L., Saiter A. (2015). Dynamic Heterogeneity and Cooperative
Length Scale at Dynamic Glass Transition in Glass Forming Liquids. Macromolecules.

[33] Wang X., Xu W.-S., Zhang H., Douglas J. F. (2019). Universal
nature
of dynamic heterogeneity in glass-forming liquids: A comparative study
of metallic and polymeric glass-forming liquids. J. Chem. Phys..

[34] Peng H., Liu H., Voigtmann T. (2022). Nonmonotonic
dynamical correlations beneath the surface
of glass-forming liquids. Phys. Rev. Lett..

[35] Appignanesi G. A. (2006). Democratic particle
motion for metabasin transitions in simple glass
formers. Phys. Rev. Lett..

[36] Hallavant K. (2024). Influence of chemical composition and structure
on the cooperative
fluctuation in supercooled glass-forming liquids. J. Phys. Chem. Lett..

[37] Donth, E. The glass transition, Springer-Verlag, Berlin, Heidelberg,(2001) p 96, p. 261ff.

[38] Colmenero J. (2015). Are polymers
standard glass-forming systems? The role of intramolecular barriers
on the glass-transition phenomena of glass-forming polymers. J. Phys.: Condens. Matter.

[39] Sasaki T., Ichimura M., Irie S. (2015). Correation
between fragility and
cooperativity in semental dynamics of glass-forming para-substituted
polystyrenes. Polymer J..

[40] Ellison C. J., Mundra M. K., Torkelson J. M. (2005). Impacts
of Polystyrene Molecular
Weight and Modification to the Repeat Unit Structure on the Glass
Transition - Nanoconfinement Effect and the Cooperativity Length Scale. Macromolecules.

[41] Alvarez F., Alegria A., Colmenero J. (1991). Relationship between the time-domain
Kohlrausch-Williams-Watts and frequency-domain Havriliak-Negami relaxation
functions. Phys. Rev. B.

[42] Saiter A. (2010). Temperature dependence
of the characteristic length scale for glassy
dynamics: Combination of dielectric and specific heat spectroscopy. Phys. Rev. E.

[43] Sato A., Sasaki T. (2018). Cooperativity of dynamics in supercooled polymeric
materials and its temperature dependence predicted from a surface
controlled model. Eur. Polym. J..

[44] Rössler E. (1991). Experimental
results revealing a change of diffusion mechanism above *T*
_g_. J. Non-Cryst. Solids.

[45] Donth E. (2002). Dynamic or
configurational approach to the glass transition. J. Non-Cryst. Solids.

[46] Garwe F. (1994). Thermal response in the splitting region of the dynamic
glass transition. J. Non-Cryst. Solids.

[47] Vogel H. (1921). Temperaturabhängigkeitsgesetz
der Viskosität von Flüssigkeiten. Phys. Z..

[48] Fulcher G.
S. (1925). Analysis
of recent measurements of the viscosity of glasses. J. Am. Ceram. Soc..

[49] Tammann G., Hesse W. (1926). Die Abhängigkeit
der Viskosität von der Temperatur
von unterkühlten Flüssigkeiten. Z. Anorg. Allg. Chem..

[50] Roland C. M. (2010). Relaxation
Phenomena in Vitrifying Polymers and Molecular Liquids. Macromolecules.

[51] Schmelzer J. W. P. (2012). Kinetic
criteria of glass formation and the pressure dependence of the glass
transition temperature. J. Chem. Phys..

[52] Schawe J. E. K. (2014). Vitrification
in a wide cooling rate range: The relations between cooling rate,
relaxation time, transition width, and fragility. J. Chem. Phys..

[53] Angell C. A. (1985). Spectroscopy
Simulation and Scattering, and the Medium Range Order Problem in Glass. J. Non-Cryst. Solids.

[54] Wang L.-M., Angell C. A., Richert R. (2006). Fragility and thermodynamics
in nonpolymeric
glass-forming liquids. J. Chem. Phys..

[55] Cavagna A. (2009). Supercooled
liquids for pedestrians. Phys. Rep..

[56] Grohens Y., Prud’homme R. E., Schultz J. (1998). Cooperativity in Backbone to Side-Chain
Conformational Rearrangements in Stereoregular PMMA. Macromolecules.

[57] Schmidt-Rohr K. (1994). Molecular Nature of the & Relaxation in Poly­(methyl methacrylate)
Investigated by Multidimensional NMR. Macromolecules.

[58] Wang X. (2012). Dynamic crossover of
the sub-Rouse modes in the glass-rubber transition
region in poly­(n-alkyl methacrylates) with different side chain lengths. Chem. Phys. Lett..

[59] Hong L., Novikov V. N., Sokolov A. P. (2011). Is there a connection
between fragility
of glass forming systems and dynamic heterogeneity/cooperativity?. J. Non-Cryst. Solids.

[60] Schawe J. E. K. (2025). The cooperativity of
atomic fluctuations in highly
supercooled glass-forming metallic melts. J.
Phys. Chem. Lett..

[61] Rössler E., Hess K.-U., Novikov V. N. (1998). Universal representation
of viscosity
in glass forming liquids. J. Non-Cryst. Solids.

[62] Kunal K. (2008). Role of chemical structure
in fragility of polymers: A quantitative
picture. Macromolecules.

[63] Mauro N. A. (2014). A structural signature
of liquid fragility. Nat. Commun..

[64] Kube S. A. (2022). Compositional dependence
of the fragility in metallic glass forming
liquids. Nat. Commun..

[65] Sokolov A. P. (2013). Cooperativity
and heterogeneity in dynamics of glass forming systems. AIP Conf. Proc..

[66] Wunderlich B. (1960). Study of the
change in specific heat of monomeric and polymeric glasses during
the glass transition. J. Phys. Chem..

[67] Schawe J. E. K. (2015). The
Gibbs free energy difference between an undercooled melt and the crystalline
phase of polymers: A new approach. J. Thermal
Anal. Calorim..

[68] Solunov H. A. (2019). Relations
between the sub-molecular and the cooperative scales in the molecular
dynamics of the glass-forming liquids. J. Non-Cryst.
Solids.

[69] Kivelson S. A. (1994). Frustration-limit clusters
in liquids. J. Chem.
Phys..

[70] Leheny R.
L. (1996). Structural
studies of an organic liquid through the glass transition. J. Chem. Phys..

[71] Tanaka H. (1999). Two-order-parameter
description of liquids. I. A general model of glass transition covering
its strong to fragile limit. J. Chem. Phys..

[72] Jaiswal A. (2016). Onset of cooperative
dynamic in an equilibrium glass-forming metallic
liquid. J. Phys. Chem. B.

[73] Corezzi S. (1999). Changes in the dynamics
of supercooled systems revealed by dielectric
spectroscopy. J. Chem. Phys..

[74] Hoffmann R., Janiak C., Kollmar C. (1991). A Chemical
Approach to the Orbitals
of Organic Polymers. Macromolecules.

[75] Dang L. X. (2000). Molecular
dynamics study of benzene-benzene and benzene-potassium ion interactions
using polarizable potential models. J. Chem.
Phys..

[76] Saida J. (2011). Atomic structure of
nanoscale quasicrystal-forming Zr-noble metal
binary metallic glasses. J. Alloys Compd..

[77] Shi Y. (2023). Revealing the relationship
between liquid fragility and medium-range
order in silicate glasses. Nat. Commun..

[78] Böhmer R., Angell C. A. (1992). Correlations of
the nonexponentiality and state dependence
of mechanical relaxations with bond connectivity in Ge-As-Se supercooled
liquids. Phys. Rev. B.

[79] Dingwell D. B. (2022). The glass transition
and the non-Arrhenian viscosity of carbonate
melts. Am. Mineral..

[80] Senkov O. N. (2007). Correlation
between fragility and glass-forming ability of metal alloys. Phys. Rev. B.

[81] Firlus A. (2022). Anisotropy-induced spin disorder in intergrown, ferrimagnetic Fe_7_S_8_ polytypes. Appl. Phys.
Lett..

[82] Beiner M., Huth H., Schröter K. (2001). Crossover
region of dynamic glass
transition: general trends and individual aspects. J. Non-Cryst. Solids.

[83] Chua Y. Z. (2017). Temperature fluctuations and the thermodynamic determination
of the
cooperativity length in glass forming liquids. J. Chem. Phys..

[84] Korus J. (1997). Temperature dependence
of *a* glass transition cooperativity. Acta Polym..

[85] Huth H. (2001). Glass transition cooperativity
from heat capacity spectroscopy —
temperature dependence and experimental uncertainties. Thermochim. Acta.

[86] Kahle S., Schröter K., Hempel E., Donth E. (1999). Calorimetric
indications
of a cooperativity onset in the crossover region of dynamic glass
transition for benzoin isobutylether. J. Chem.
Phys..

[87] Puente J.
A. S. (2015). Segmental
mobility and glass transition of poly­(ethylene-vinyl
acetate)­copolymers: Is there a continuum in the dynamic glass transitions
from PVAc to PE?. Polymer.

[88] Rijal B. (2016). Correlated and cooperative
motions in segmental relaxation: Influence
of constitutive unit weight and intermolecular interactions. Phys. Rev. E.

[89] Kahle S. (1997). Glass-Transition Cooperativity Onset in a Series of Random Copolymers
Poly­(*n*-butyl methacrylate-*stat*-styrene). Macromolecules.

[90] Fosse C. (2022). Cooperativity and fragility in furan-based polyesters with different
glycolic subunits as compared to their terephthalic counterparts. J. Non-Cryst. Solids.

[91] Ferry J. D. (1957). Dynamic mechanical properties of polyethyl methacrylate. J. Colloid Sci..

[92] Bourdet A. (2021). Molecular mobility in amorphous biobased copolyesters
obtained with
2,5- and 2,4-furandicarboxylate acid. Polymer.

[93] Chua Y. Z. (2014). Glass transition cooperativity
from broad band heat capacity spectroscopy. Colloid Polym. Sci..

[94] Schawe J. E. K., Hess K.-U. (2019). The kinetics of the glass transition
of silicate glass
measured by fast scanning calorimetry. Thermochim.
Acta.

