# Supplementary material for: The Universality of Cooperative Fluctuations in Glass-Forming Supercooled Liquids
Source: J Phys Chem Lett. 2025 Nov 17;16(47):12255–65. doi: 10.1021/acs.jpclett.5c03185 (PMC12670492; doi:10.1021/acs.jpclett.5c03185)
Supplement: Supplementary file 1 [file jz5c03185_si_001.pdf]

Supporting Information

The universality of cooperative fluctuation in glass-forming supercooled liquids

Jürgen E. K. Schawe[1*], Kylian Hallavant[2], Antonella Esposito[2], Jörg F. Löffler[1*], Allisson Saiter-Fourcin[2*]

[1] Laboratory of Metal Physics and Technology, Department of Materials, ETH Zurich, 8093 Zurich, Switzerland

[2] Université de Rouen Normandie, INSA Rouen Normandie, CNRS, Groupe de Physique des Matériaux UMR 6634, 76000 Rouen, France

* Corresponding authors

First corresponding author: Jürgen E.K. Schawe, juergen.schawe@mat.ethz.ch
Co-corresponding authors:
Jörg F. Löffler, joerg.loeffler@mat.ethz.ch
Allisson Saiter-Fourcin, allison.saiter@univ-rouen.fr

S1. Consistency test of the used data

To check for the consistency of the kinetic data, the fragility index was determined from the VFTH parameters:

$$m_{\mathrm{VFTH}} = \frac{B\,T_g}{(T_g - T_v)^2} \tag{S1}$$

The values of m_{VFTH} calculated in this work were then plotted versus the values of the kinetic fragility index m reported in the literature (Fig. S1). Only for the materials lying on the blue line passing through the origin of the coordinate system and having a slope equal to 1 are the VFTH parameter consistent with the reported fragility index. The other data (indicated by numbers in Fig. S1, with the details reported in Table 1 and Table 2 in the manuscript) show significant deviations from this consistency line. These deviations are most likely related to the uncertainty in the determination of m from measurements obtained in a relatively small range. This inconsistency led to increased errors in Figs. 2a, 3 and 4 in the manuscript.

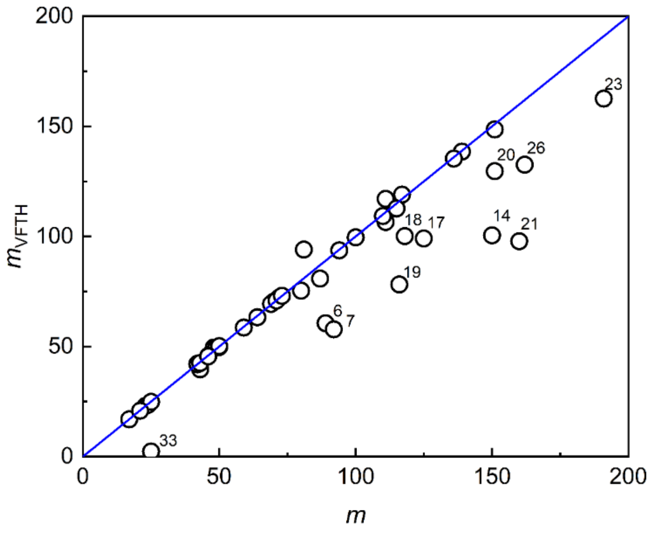

Figure S1. Consistency test of the reported data of the VFTH parameters and the fragility indices. Comparison of the values of the fragility index m_{VFTH}, calculated from the reported VFTH parameters B, T_v and T_g using Eq. (S1), with the reported values of the dynamic fragility index m from the literature.

Table S1. Sample names and individual fit results of the data in Fig. 1 (manuscript), using Eq. (8) (manuscript).

Number	Material	Name	ξ_0 [nm]	$\xi(T_g)$ [nm]
1	**PPglycol**	Poly (propylene glycol)	0.61	3.43
2	**PIB**	Poly isobutylene	0.27	2.87
3	**Glycerol**		0.47	3.27
4	**SBR**	Styrene butadiene rubber with 23 wt% styrene	0.29	3.62
5	**BIBE**	Benzoin isobutylether	0.29	4.41
6	**EVA_60**	Ethylene-vinyl-acetate with 60 % vinyl acetate groups	0.30	2.75
7	**EVA_80**	Ethylene-vinyl-acetate with 80 % vinyl acetate groups	0.31	3.23
8	**PnBMA2%**	Copolymer of n-butyl methacrylate with 2 wt% styrene	0.09	2.17
9	**PnBMA19%**	Copolymer of n-butyl methacrylate with 19 wt% styrene	0.16	2.22
10	**Se**	Selenium	0.28	3.12
11	**PVAc**	Poly vinyl acetate	0.24	2.91
12	**PBF**	Poly (butylene furanoate)	0.28	3.62
13	**PEMA**	Poly (ethyl methacrylate)	0.20	2.04
14	**PPT**	Poly (propylene yerephthalate)	0.25	3.10
15	**PPF**	Poly (propylene furanoate)	0.22	3.34
16	**PLA**	Poly (lactic acid)	0.22	3.66
17	**2,4-PEF**	Poly (ethylene 2,4-furandicarboxylate)	0.25	3.46
18	**PE-2,4-2,5-F**	Poly (ethylene 2,4-*co*-2,5-furandicarboxylate)	0.26	4.20
19	**2,5-PEF**	Poly (ethylene 2,5-furandicarboxylate)	0.32	3.13
20	**PETg**	Glycol-modified poly (ethylene terephthalate)	0.14	3.58
21	**PET**	Poly (ethylene terephthalate)	0.20	3.74
22	**PMMA**	Poly (methyl methacrylate)	0.21	1.43
23	**PVC**	Poly (vinyl chloride)	0.18	2.20
24	**PS**	Polystyrene	0.20	3.13
25	**PC**	Polycarbonate	0.17	3.98
26	**PEI**	Poly (ethylene imine)	0.18	3.48
27	**Pt-based glass**	$Pt_{57.4}Cu_{14.7}Ni_{5.3}P_{22.6}$	0.40	2.80
28	**Pd-based glass**	$Pd_{43}Cu_{27}Ni_{10}P_{20}$	0.35	2.65
